# AQP4-IgG-positive myelitis occurring in a patient with small cell lung cancer: a case report

**DOI:** 10.3389/fonc.2026.1867559

**Published:** 2026-07-29

**Authors:** Meijian Yang, Dongjie Du, Xiaoci Cao, Jia Liu, Jing Zhao

**Affiliations:** 1 Oncology Department II, Hebei General Hospital, Shijiazhuang, China; 2 Vascular Surgery Department, Hebei General Hospital, Shijiazhuang, China

**Keywords:** acute myelopathy, AQP4-IgG, neuromyelitis optica spectrum disorder, small cell lung cancer, spinal cord metastasis mimic

## Abstract

**Background:**

Acute myelopathy in patients with small cell lung cancer (SCLC) is often attributed to metastatic spinal cord involvement. However, non-compressive inflammatory conditions, particularly aquaporin-4 immunoglobulin G (AQP4-IgG)-positive neuromyelitis optica spectrum disorder (NMOSD), can produce similar clinical and radiologic features, creating substantial diagnostic uncertainty.

**Case presentation:**

A male aged 65 years with extensive-stage SCLC presented with acute urinary retention followed by rapidly progressive paraplegia. Spinal magnetic resonance imaging (MRI) revealed longitudinally extensive intramedullary T2-hyperintense lesions affecting the cervical and thoracic spinal cord, with patchy contrast enhancement and no evidence of vertebral destruction, epidural mass, or spinal cord compression. Cerebrospinal fluid (CSF) examination demonstrated inflammatory abnormalities without malignant cells, and infectious causes were excluded. Serum and CSF testing were strongly positive for AQP4-IgG, establishing a diagnosis of AQP4-IgG-positive NMOSD. The patient received high-dose intravenous methylprednisolone followed by adjunctive intravenous immunoglobulin (IVIG). Plasma exchange was not performed because of advanced malignancy and concerns regarding procedural risks. Satralizumab was initiated during corticosteroid tapering for maintenance immunotherapy. Neurological function subsequently stabilized, and partial recovery of independence in activities of daily living was achieved.

**Conclusions:**

This case highlights that AQP4-IgG-positive myelitis can occur in patients with SCLC. In patients presenting with non-compressive longitudinally extensive transverse myelitis (LETM), particularly when imaging shows no evidence of compression and CSF cytology is negative for malignant cells, prompt AQP4-IgG testing is critical to prevent misdiagnosis and enable early initiation of appropriate immunotherapy.

## 1 Introduction

Acute myelopathy in patients with small cell lung cancer (SCLC) is commonly attributed to metastatic spinal cord involvement and frequently prompts urgent oncologic treatment. However, spinal cord lesions in this population are not always metastatic. Non-compressive inflammatory disorders, including neuromyelitis optica spectrum disorder (NMOSD), may produce similar clinical and radiologic manifestations, creating substantial diagnostic challenges. NMOSD is an autoimmune astrocytopathy characterized by longitudinally extensive transverse myelitis (LETM) and aquaporin-4 immunoglobulin G (AQP4-IgG) seropositivity (1, 2).

Although rare, AQP4-IgG-positive NMOSD has been reported in patients with various cancers, including lung cancer (3–5). In patients with advanced cancer, its clinical and radiologic features may closely resemble intramedullary metastasis, increasing the likelihood of misdiagnosis and inappropriate treatment. Failure to recognize NMOSD may result in unnecessary metastasis-directed interventions and delayed initiation of immunotherapy, which is essential for optimizing neurological outcomes. Therefore, NMOSD should be considered in patients with atypical or non-compressive spinal cord lesions, and early AQP4-IgG testing is critical for establishing an accurate diagnosis (6).

This report describes a case of AQP4-IgG-positive myelitis in a patient with extensive-stage SCLC that was initially suspected to represent spinal metastasis, emphasizing an important diagnostic pitfall and the value of early immunologic evaluation.

## 2 Case report

A male aged 65 years with extensive-stage SCLC presented with acute urinary retention followed by rapidly progressive bilateral lower-extremity weakness that advanced to complete paraplegia within several days. His medical history included hypertension, coronary artery disease, sick sinus syndrome, atrial fibrillation, and type 2 diabetes mellitus.

**Figure 1:**
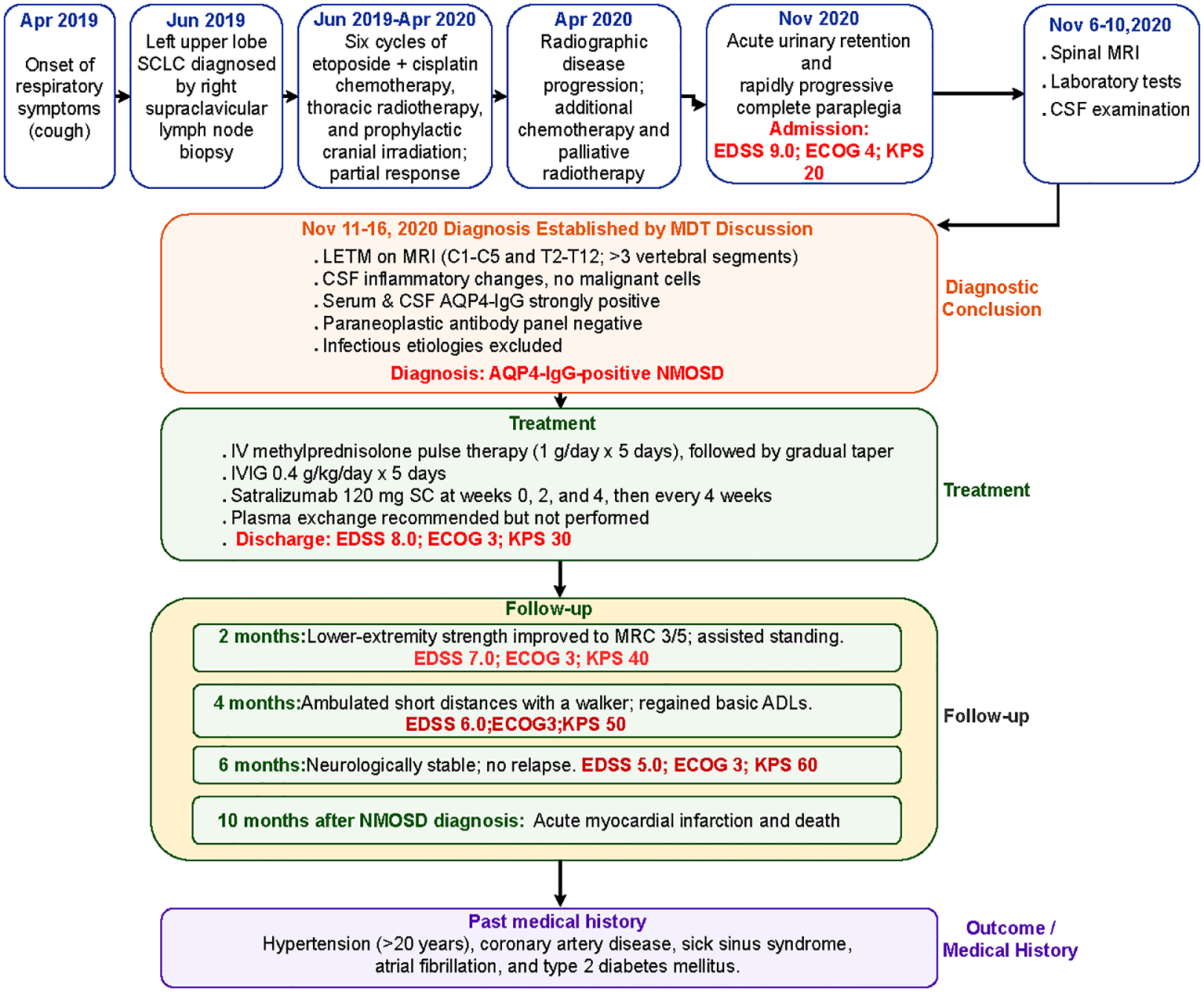
Patient’s clinical course timeline. ADLs, activities of daily living; AQP4-IgG, aquaporin-4 immunoglobulin G; C1–C5, cervical spinal cord segments 1–5; CSF, cerebrospinal fluid; ECOG, Eastern Cooperative Oncology Group performance status; EDSS, Expanded Disability Status Scale; IV, intravenous; IVIG, intravenous immunoglobulin; KPS, Karnofsky Performance Status; LETM, longitudinally extensive transverse myelitis; MDT, multidisciplinary team; MRC, Medical Research Council; MRI, magnetic resonance imaging; NMOSD, neuromyelitis optica spectrum disorder; SC, subcutaneous; SCLC, small-cell lung cancer; T2–T12, thoracic spinal cord segments 2–12.

The patient initially developed respiratory symptoms, including cough, in April 2019. In June 2019, biopsy of a right supraclavicular lymph node confirmed left upper lobe SCLC. The clinical course of the patient is summarized in **Figure 1**. First-line treatment consisted of six cycles of etoposide plus cisplatin, thoracic radiotherapy, and prophylactic cranial irradiation, resulting in a partial response. Radiographic progression was documented in April 2020, after which additional chemotherapy and palliative radiotherapy were administered.

Approximately 10 days before admission, the patient developed progressive urinary dysfunction, initially manifested by difficulty voiding and subsequently progressing to complete urinary retention. Concurrently, bilateral lower-extremity weakness rapidly worsened, ultimately leading to complete paraplegia. These neurologic symptoms occurred in November 2020, approximately 17 months after the diagnosis of SCLC. On admission, the patient was alert but severely disabled, with a Karnofsky Performance Status (KPS) score of 20 (**Figure 1**). Neurologic examination revealed flaccid paraplegia, Medical Research Council (MRC) grade 0/5 strength in both lower extremities, a sensory level below T8, absent lower-limb deep tendon reflexes, reduced anal sphincter tone, and marked bladder distension.

**Figure 2:**
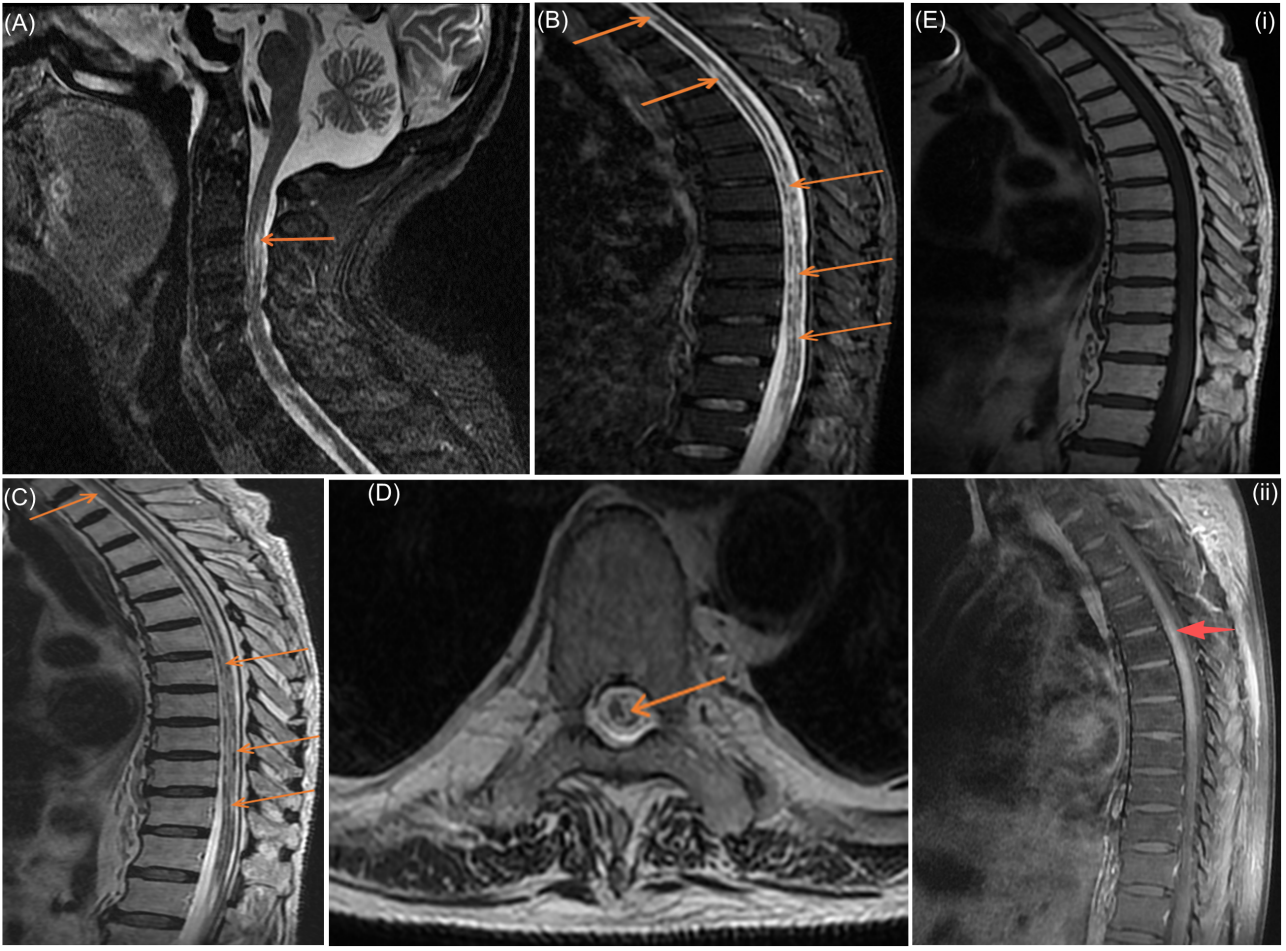
Spine MRI findings. **(A–C)** Sagittal cervical and thoracic MRI showed longitudinally extensive intramedullary lesions from C1 to C5 and T2 to T12, without vertebral involvement or spinal cord compression. **(D)** Axial T2-weighted imaging showed patchy intramedullary hyperintensity. **(E)** Pre-contrast sagittal T1-weighted imaging (T1WI) showed no definite enhancement (i), whereas contrast-enhanced sagittal T1WI showed linear peripheral spinal cord enhancement (ii).

Laboratory testing demonstrated mild leukocytosis, anemia, thrombocytopenia, hyponatremia, and elevated tumor markers, including neuron-specific enolase (NSE) and pro-gastrin-releasing peptide (Pro-GRP). Spinal magnetic resonance imaging (MRI) revealed longitudinally extensive intramedullary lesions involving the cervical cord from C1 to C5 and the thoracic cord from T2 to T12. The lesions appeared as multiple patchy hyperintensities on cervical and thoracic STIR images (**Figure 2A, B**) and as intramedullary hyperintensities on sagittal and axial T2-weighted images (**Figure 2C, D**). Contrast-enhanced T1-weighted imaging demonstrated linear marginal enhancement of the spinal cord lesions compared with the non-contrast images [**Figure 2E** (i) and (ii)]. No vertebral destruction or epidural compression was identified. Based on these findings, intramedullary spinal metastasis was initially suspected.

**Figure 3:**
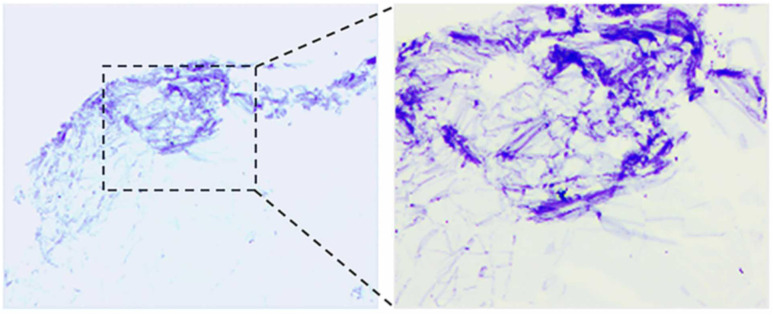
Cerebrospinal fluid cytology. CSF cytology demonstrated inflammatory changes without malignant cells.

Cerebrospinal fluid (CSF) analysis demonstrated elevated protein levels and inflammatory pleocytosis without malignant cells. Infectious causes were excluded. Given the non-compressive pattern of spinal cord involvement and negative CSF cytology, multidisciplinary assessment favored inflammatory myelitis over metastatic spinal cord disease (**Figure 3**). Subsequent immunologic testing demonstrated strongly positive AQP4-IgG in both serum and CSF, whereas a comprehensive paraneoplastic antibody panel was negative. Collectively, the clinical, radiologic, and immunologic findings established a diagnosis of AQP4-IgG-positive NMOSD.

Treatment was initiated immediately after diagnosis using an integrated acute-phase immunotherapy strategy. Given the severity of the AQP4-IgG-positive myelitis, high-dose intravenous methylprednisolone pulse therapy was administered at 1 g/day for 5 consecutive days, followed by stepwise tapering with intravenous methylprednisolone at 500 mg/day for 3 days, 240 mg/day for 3 days, and 120 mg/day for an additional 3 days. The regimen was then transitioned to oral prednisone at 60 mg/day for 7 days and 50 mg/day for 7 days, followed by gradual tapering to 30–40 mg/day and finally to a maintenance dose of 5–10 mg/day. The total duration of corticosteroid therapy was approximately 6 months. Because lower-extremity strength showed only limited improvement after steroid pulse therapy, adjunctive IVIG was administered at 0.4 g/kg/day for 5 consecutive days. Plasma exchange was also recommended as a potential escalation therapy to remove circulating pathogenic antibodies; however, it was not performed because the patient’s family declined the procedure due to advanced malignancy and concerns regarding procedural risks. During corticosteroid tapering, satralizumab was initiated for long-term relapse prevention, given the high relapse risk associated with AQP4-IgG-positive NMOSD. Satralizumab was administered subcutaneously at 120 mg at weeks 0, 2, and 4, followed by maintenance dosing every 4 weeks.

Serial assessments using the Expanded Disability Status Scale (EDSS), Eastern Cooperative Oncology Group (ECOG) performance status, and KPS were conducted at admission, discharge, and follow-up. These evaluations demonstrated progressive improvement in neurologic disability and overall functional status during treatment and maintenance immunotherapy (**Figure 1**).

After 2 weeks of hospitalization, the patient exhibited partial neurologic recovery and was discharged. At discharge, voluntary movement had returned in both lower extremities, with muscle strength improving to approximately MRC grade 2. He was able to sit independently. Urinary retention had improved, although intermittent catheterization remained necessary. Oral corticosteroids were gradually tapered, and outpatient rehabilitation was initiated.

At the 2-month follow-up, lower-extremity strength had improved to MRC grade 3, allowing assisted standing. Sensory impairment had diminished, and urinary function remained stable with self-administered intermittent catheterization and no urinary tract infection. By 4 months, the patient was able to walk short distances with a walker and had regained basic activities of daily living. Nevertheless, persistent lower-extremity weakness and fatigue prevented him from returning to work (**Figure 1**).

At the 6-month follow-up, neurologic status remained stable during corticosteroid tapering and ongoing satralizumab therapy, with no evidence of relapse. Serial neurologic examinations, spinal MRI, and oncologic evaluations revealed no new spinal cord compression or central nervous system metastasis. Systemic oncologic reassessment, including chest, abdominal, and pelvic imaging, showed no evidence of additional primary malignancy. During satralizumab therapy, serial laboratory monitoring, including inflammatory markers such as C-reactive protein and procalcitonin, together with chest imaging, revealed no evidence of new infection, opportunistic infection, or tumor progression (**Figure 1**).

At the 10-month follow-up after NMOSD diagnosis, the patient remained neurologically stable without relapse. However, he subsequently developed sudden chest pain and presented to the emergency department. Electrocardiography demonstrated sinus rhythm, ST-segment elevation in leads I, aVL, and V2–V6, pathologic Q waves, and reciprocal ST-segment depression in leads II, III, and aVF. Cardiac troponin and myocardial enzyme levels were elevated, and echocardiography indicated acute cardiac dysfunction. The patient was diagnosed with coronary atherosclerotic heart disease, acute extensive anterior myocardial infarction, Killip class II, and acute heart failure.

Although coronary angiography and thrombolytic therapy were planned, the patient experienced sudden cardiopulmonary arrest, most likely secondary to cardiogenic shock, and died despite resuscitative efforts. Notably, the patient had substantial preexisting cardiovascular comorbidities, including hypertension, coronary artery disease, sick sinus syndrome, and atrial fibrillation.

## 3 Discussion

This case demonstrates that acute spinal cord lesions in patients with SCLC should not automatically be attributed to metastatic disease solely because of an underlying cancer diagnosis. In the present patient, metastatic spinal cord involvement was initially considered highly likely because of extensive-stage SCLC and rapidly progressive paraplegia. However, several clinical and radiologic findings were inconsistent with metastatic spinal cord compression. Spinal MRI revealed longitudinally extensive intramedullary T2-hyperintense lesions involving both the cervical and thoracic spinal cord, accompanied by patchy enhancement but without vertebral destruction, epidural mass formation, or definitive compressive pathology. CSF analysis showed inflammatory abnormalities without malignant cells, and infectious causes were excluded. Subsequent testing demonstrated strong AQP4-IgG positivity in both serum and CSF, supporting a diagnosis of AQP4-IgG-positive NMOSD. According to the 2015 International Consensus Diagnostic Criteria for NMOSD, the presence of AQP4-IgG together with a core clinical characteristic, such as acute myelitis, is sufficient for diagnosis, and LETM represents one of the most characteristic radiologic features of the disorder (2).

The imaging findings are particularly relevant because metastatic spinal cord disease and NMOSD-associated myelitis require distinct therapeutic approaches. Metastatic spinal cord compression is most commonly caused by vertebral metastases with epidural extension and typically manifests as vertebral destruction, epidural soft-tissue mass formation, and spinal cord compression or displacement (7). By contrast, intramedullary spinal cord metastases usually appear as solitary or multiple enhancing lesions with surrounding edema rather than continuous longitudinally extensive lesions (7). NMOSD-associated myelitis characteristically presents as longitudinally extensive intramedullary T2-hyperintense lesions extending across three or more vertebral segments, frequently involving the central spinal cord (2). Therefore, in patients with cancer presenting with non-compressive LETM and negative CSF cytology, early AQP4-IgG testing should be incorporated into the diagnostic evaluation to minimize misdiagnosis and facilitate timely initiation of immunotherapy (8–10).

Several reports have described AQP4-IgG-positive NMOSD in patients with cancer (8–10). However, substantial heterogeneity exists regarding the timing of neurologic symptom onset relative to cancer diagnosis and the spectrum of clinical manifestations. Deuel and Bunch reported a patient who developed LETM and optic neuritis before the diagnosis of SCLC (11). Baik et al. described a patient with metastatic lung adenocarcinoma who developed LETM followed by optic neuritis, with AQP4 immunoreactivity detected in metastatic lymph node tissue (12). In contrast, the present patient developed NMOSD approximately 17 months after the diagnosis of SCLC, and LETM remained the dominant manifestation throughout the clinical course without optic neuritis or other core NMOSD syndromes. Notably, the initial presentation consisted of acute urinary retention and rapidly progressive paraplegia, closely resembling spinal metastasis or metastatic spinal cord compression.

Heterogeneity in tumor-associated immune responses may influence both the anatomical distribution and clinical manifestations of NMOSD (8, 10). Variations in tumor burden, antitumor immune activity, and AQP4-IgG levels may produce distinct patterns of central nervous system injury, resulting in spinal cord involvement in some patients and combined optic nerve and spinal cord involvement in others. However, evidence linking these factors to specific NMOSD phenotypes remains limited. Although this patient had strongly positive serum and CSF AQP4-IgG results, serial antibody measurements and detailed immunological studies were unavailable, preventing evaluation of potential associations between the clinical phenotype and antibody levels or immune response characteristics. Furthermore, ectopic expression of AQP4 or related antigens within tumor tissue has been proposed as a trigger for cross-reactive autoimmunity and subsequent AQP4-IgG production (9, 10). However, because tumor tissue was unavailable in the present case, this mechanism could not be evaluated and should not be inferred. Overall, this case highlights the clinical heterogeneity and diagnostic complexity of AQP4-IgG-positive NMOSD occurring in patients with cancer, while emphasizing the need for larger studies to clarify the determinants of specific phenotypes.

Treatment decisions also presented significant challenges. High-dose intravenous corticosteroids remain the standard first-line treatment for acute NMOSD attacks, whereas plasma exchange is generally recommended for severe attacks or inadequate therapeutic responses (2). The patient received high-dose intravenous methylprednisolone; however, plasma exchange was declined by the family because of concerns regarding advanced malignancy and procedural risks. Intravenous immunoglobulin was therefore administered as adjunctive therapy. Given the high relapse risk associated with AQP4-IgG-positive NMOSD, satralizumab was initiated during corticosteroid tapering as maintenance immunotherapy. Satralizumab, an interleukin-6 (IL-6) receptor inhibitor, has been shown to reduce relapse risk in patients with AQP4-IgG-positive NMOSD (13). Nevertheless, its use in patients with concomitant malignancy requires careful consideration. The IL-6 signaling pathway contributes to inflammation, B-cell activation, blood-brain barrier dysfunction, and AQP4-IgG production, providing a biological rationale for IL-6 receptor blockade. At the same time, evidence regarding the safety of satralizumab in patients with active or advanced malignancies remains limited. Therefore, close monitoring for infections, opportunistic infections, and tumor progression is warranted. In this case, serial laboratory testing, inflammatory markers, and chest imaging during follow-up showed no evidence of new infections or tumor progression.

During follow-up, the patient achieved partial neurological recovery and remained relapse-free. Serial neurological examinations, spinal MRI studies, and oncological assessments demonstrated no evidence of recurrent spinal cord compression or central nervous system metastases. However, assessment of long-term relapse prevention was limited because the patient died from an acute extensive anterior-wall myocardial infarction 10 months after the diagnosis of NMOSD. This outcome emphasizes that prognosis in patients with NMOSD and malignancy depends not only on neurological disease control but also on tumor burden, baseline organ function, and systemic comorbidities.

This case report has several limitations. First, the original tumor specimen was unavailable for AQP4 immunohistochemical evaluation, precluding confirmation of a direct biological association between SCLC and NMOSD. Second, as a single-case report, this study cannot establish relationships among tumor burden, AQP4-IgG titers, immune response characteristics, and clinical phenotype. Finally, long-term follow-up was restricted by the patient’s death from acute myocardial infarction.

In conclusion, when patients with SCLC present with acute non-compressive LETM, spinal metastasis should not be presumed solely on the basis of a cancer history. In the absence of radiological evidence of compression or malignant cells on CSF cytology, early AQP4-IgG testing is essential for identifying AQP4-IgG-positive NMOSD, avoiding unnecessary cancer-directed interventions, and facilitating timely initiation of immunotherapy.

## Data Availability

The raw data supporting the conclusions of this article will be made available by the authors, without undue reservation.
